# Cellular re- and de-programming by microenvironmental memory: why short TGF-β1 pulses can have long effects

**DOI:** 10.1186/1755-1536-6-12

**Published:** 2013-06-19

**Authors:** Ariel Bing-Shi Tan, Sebastian Kress, Leticia Castro, Allan Sheppard, Michael Raghunath

**Affiliations:** 1 NUS Graduate School for Integrative Sciences and Engineering, National University of Singapore, 28 Medical Drive, Singapore; 2 NUS Tissue Engineering Programme, Life Science Institute, National University of Singapore, 28 Medical Drive, Singapore 117456; 3 Department of Bioengineering, Faculty of Engineering, National University of Singapore, 9 Engineering Drive 1, Singapore 117576; 4 Faculty of Biology, Bayerische Julius-Maximilians-Universität Würzburg, Sanderring 2, Würzburg 97070Germany; 5 Liggins Institute, University of Auckland, 85 Park Road, Auckland 1023New Zealand; 6 Department of Biochemistry, Yong Loo Lin School of Medicine, National University of Singapore, 8 Medical Drive, Singapore 117597

**Keywords:** Fibrosis, Transforming growth factor-beta 1, Extracellular matrix, Memory, Pulses, Phenotype, Kinetics, Cytokine

## Abstract

**Background:**

Fibrosis poses a substantial setback in regenerative medicine. Histopathologically, fibrosis is an excessive accumulation of collagen affected by myofibroblasts and this can occur in any tissue that is exposed to chronic injury or insult. Transforming growth factor (TGF)-β1, a crucial mediator of fibrosis, drives differentiation of fibroblasts into myofibroblasts. These cells exhibit α-smooth muscle actin (α-SMA) and synthesize high amounts of collagen I, the major extracellular matrix (ECM) component of fibrosis. While hormones stimulate cells in a pulsatile manner, little is known about cellular response kinetics upon growth factor impact. We therefore studied the effects of short TGF-β1 pulses in terms of the induction and maintenance of the myofibroblast phenotype.

**Results:**

Twenty-four hours after a single 30 min TGF-β1 pulse, transcription of fibrogenic genes was upregulated, but subsided 7 days later. In parallel, collagen I secretion rate and α-SMA presence were elevated for 7 days. A second pulse 24 h later extended the duration of effects to 14 days. We could not establish epigenetic changes on fibrogenic target genes to explain the long-lasting effects. However, ECM deposited under singly pulsed TGF-β1 was able to induce myofibroblast features in previously untreated fibroblasts. Dependent on the age of the ECM (1 day versus 7 days’ formation time), this property was diminished. *Vice versa*, myofibroblasts were cultured on fibroblast ECM and cells observed to express reduced (in comparison with myofibroblasts) levels of collagen I.

**Conclusions:**

We demonstrated that short TGF-β1 pulses can exert long-lasting effects on fibroblasts by changing their microenvironment, thus leaving an imprint and creating a reciprocal feed-back loop. Therefore, the ECM might act as mid-term memory for pathobiochemical events. We would expect this microenvironmental memory to be dependent on matrix turnover and, as such, to be erasable. Our findings contribute to the current understanding of fibroblast induction and maintenance, and have bearing on the development of antifibrotic drugs.

## Background

Tissue repair is a physiological response to tissue damage. It starts with cell infiltration and inflammation at the site of the lesion, progresses with the formation of extracellular matrix (ECM) and ends with its remodeling, leaving a localized scar. When this response is triggered repeatedly or perpetuated, fibrosis ensues. The worldwide clinical burden of fibrosis is substantial with at least 5 million cases of idiopathic lung fibrosis [1], and 170 million people with chronic hepatitis C at risk for liver cirrhosis [2]. Fibrosis around implants can effectively sequester them from surrounding tissue and impede their function [3-6]. Amongst various cytokines implicated in fibrosis, transforming growth factor-β1 (TGF-β1) is the most notorious. It facilitates the differentiation of fibroblasts, hepatic stellate cells [7], fibrocytes [8], and epithelial cells [9] into myofibroblasts, the drivers of collagen deposition and tissue contraction. After fulfilling their initial repair task myofibroblasts undergo apoptosis. In pathological conditions, however, activated myofibroblasts persist and drive the extent of the fibrotic process creating a surplus of collagenous scar tissue [10]. Current consensus identifies myofibroblasts through a combination of three markers, namely, collagen I secretion, contractile protein α-smooth muscle actin (α-SMA) and the cytoskeletal component F-actin [11].

In contrast to hormones, growth factors (GF) like TGF-β1 act locally due to their short half-lives, slow diffusion rates and range [12]. Although very little is known about whether active TGF-β1 levels in tissue are locally elevated for minutes, hours, or longer. So far, only one study has addressed this question and demonstrated that active TGF-β1 levels in healing skin wounds indeed fluctuate at least on a daily basis [13]. This supports the notion that pulsatile regulation pervades most *in vivo* physiological systems. Typical examples are hormones that are released in bursts of 30 to 90 min intervals like the gonadotropin-releasing hormone [14] or cortisol [15]. Accordingly, hormone-responsive cells are conditioned to respond to repetitive pulses and their frequency. There is growing evidence that the duration of GF impact also modulates cell response. Multiple 24 h pulses of platelet-derived growth factor applied in 7 day intervals drives osteoblastic differentiation of pre-osteoblasts while continuous application is inhibitory [16]; cell cycle commitment achieved with 10 h of continuous exposure to platelet-derived growth factor can be replaced by two short pulses (minimum 30 min over an 8 h interval period) [17]; a 1 min single pulse of nerve growth factor can trigger long-term neuronal excitability [18]. Current *in vitro* models of fibrosis rely on the continuous exposure of cells to 2 to 20 ng/ml of recombinant TGF-β1 for 3 to 5 days to generate myofibroblasts. Here, we studied single and double TGF-β1 pulses with regard to induction and maintenance of the myofibroblast phenotype and observed long-lasting effects that can be explained by a cascade of matrix deposition and GF storage events, which highlights the ECM as a pericellular memory system.

## Results

### A 0.5 h TGF-β1 pulse was sufficient to effects changes for up to 7 days

**Figure 1:**
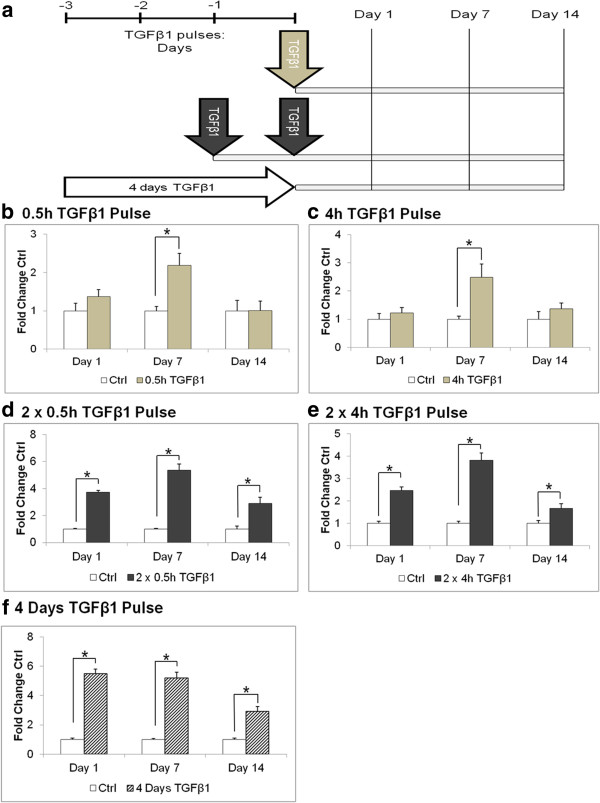
**A TGF-β1 pulse(s) elevated the collagen I secretion rate. (a)** Growth-arrested fibroblasts were treated with or without TGF-β1 according to the cell culture setup comparing single and double TGF-β1 pulses with the traditional 4 days of TGF-β1 treatment. Normalised densitometric SDS-PAGE analysis of the 24 h collagen secretion rate of pulsed fibroblasts for the **(b)** 0.5 h, **(c)** 4 h; **(d)** 2 × 0.5 h, **(e)** 2 × 4 h TGF-β1 pulses, and **(f)** 4 days of TGF-β1 treatment. *P <0.05 versus respective untreated controls. Data are represented as mean ± SD, calculated from three independent studies in triplicate, and expressed as fold changes over respective controls. SD, standard deviation; TGF-β1 transforming growth factor-β1.

**Figure 2:**
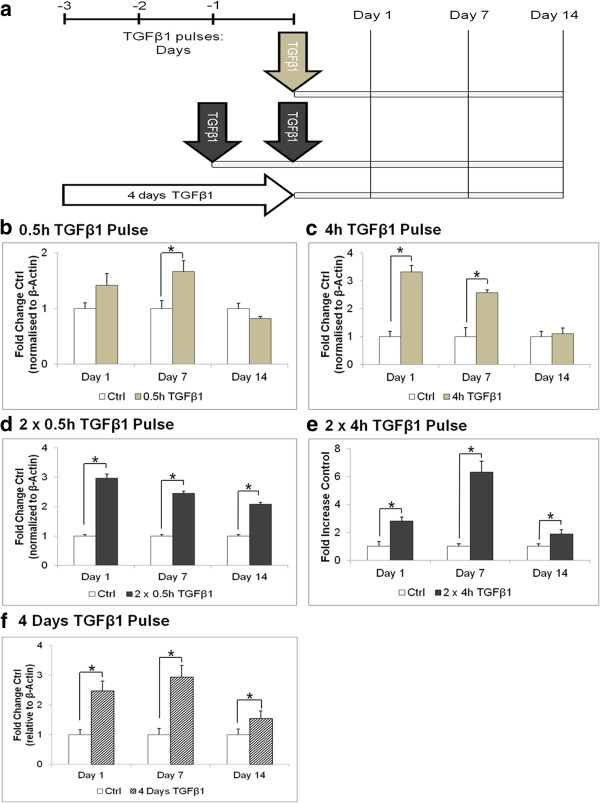
**A TGF-β1 pulse(s) elevated α-SMA expression. (a)** Growth-arrested fibroblasts were treated with or without TGF-β1 according to the cell culture setup comparing single and double TGF-β1 pulses with the traditional 4 days of TGF-β1 treatment. Densitometric analysis of α-SMA immunoblots normalised to β-actin bands for the **(b)** 0.5 h, **(c)** 4 h; **(d)** 2 × 0.5 h, **(e)** 2 × 4 h, and (**f**) 4 days of TGF-β1 treatment. *P <0.05 versus respective untreated controls. Data are represented as mean ± SD, calculated from three independent studies in triplicate, and expressed as fold changes over respective controls. α-SMA, α-smooth muscle actin; SD, standard deviation; TGF-β1 transforming growth factor-β1.

We present novel evidence that a pulse of TGF-β1, ranging from a mere 0.5 h to 4 h, elevated collagen I secretion rate sampled over a 24 h period and increased α-SMA expression. Results demonstrate the creation and maintenance of the myofibroblast phenotype 7 days post-pulse, with reversion to baseline 14 days post-pulse. An additional pulse, administered 24 h later potentiated the maintenance of the myofibroblast phenotype. In comparison with traditional exposure to 4 days of TGF-β1 treatment, we observed that double TGF-β1 pulses displayed a profile similar to fibroblasts treated with 4 days of TGF-β1 exposure (Figure 1, Figure 2, Additional file 1: Figure S1, Additional file 2: Figure S2 and Additional file 3: Figure S3).

### Fibrogenic genes were upregulated 24 h post-TGF-β1 pulse

To better characterise the long-lasting effects observed with single and double TGF-β1 pulse(s), transcription of selected fibrogenic genes relevant for the TGF-β1 signalling and collagen regulation pathway was monitored. mRNA levels of ACTA2 (α-SMA); TGF-β1 signalling molecule, frizzled-8 (FZD8); reactive oxygen species product, NADPH oxidase 4 (NOX4) and regulator of matrix proteins, transmembrane glycoprotein tetraspanin 2 (TSPAN2) were significantly elevated 24 h after a single TGF-β1 pulse for up to 7 days. Double pulses doubled the duration of this effect (Table 1).

**Table 1:** Selected fibrosis-related genes were upregulated post-TGF-β1 pulse

**Model**	**Single pulse - 0.5 h, 4 h**			**Double pulse - 2** × **0.5 h, 2** × **4 h**		
**Gene/day**	**1**	**7**	**14**	**1**	**7**	**14**
**α-SMA**	20 ± 2.6*	1.3 ± 0.4	0.8 ± 0.2	20 ± 2.9*	5.6 ± 1.3*	1.2 ± 0.2
**FZD8**	90 ± 25*	2 ± 0.8*	1.1 ± 0.2	100 ± 30*	25 ± 11*	2.3 ± 1.5
**NOX4**	50 ± 25*	1.4 ± 0.4	0.9 ± 0.1	74 ± 29*	9.3 ± 2.5*	1.6 ± 0.4
**TSPAN2**	500 ± 100*	3 ± 1.5*	1.5 ± 0.3	240 ± 70*	20 ± 7.8*	2.9 ± 1.1*

Fibroblasts were pulsed with or without TGF-β1 at the indicated time points. The expression of selected genes was quantified by RT-PCR analysis. Data are represented as averages of fold changes of TGF-β1 pulse-induced gene expression ± SD, quantified from triplicate studies in duplicate, and expressed as fold changes over respective controls (rounded to 1 dp). *P <0.05 versus respective untreated controls. *α-SMA* α-smooth muscle actin, *FZD8* frizzled family receptor 8, *NOX4* NADPH oxidase 4, *TSPAN2* transmembrane glycoprotein tetraspanin 2.

### Single TGF-β1 pulses triggered sustained autocrine TGF-β1 production

**Figure 3:**
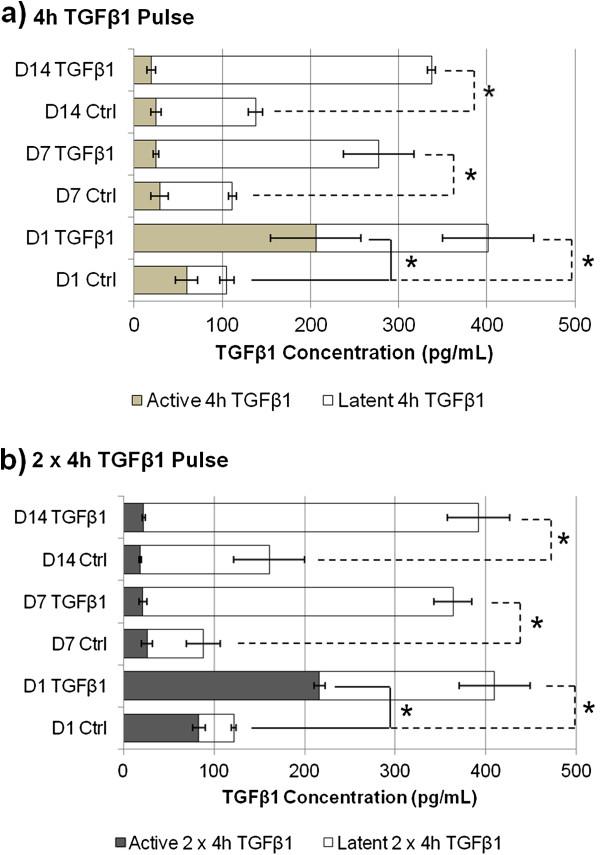
**Exogenous TGF-β1 pulses induced autocrine TGF-β1 production.** Active TGF-β1 in single and double pulses was elevated in culture medium only 24 h after **(a)** 4 h and **(b)** 2 × 4 h TGF-β1 pulse(s). Acidic activation of latent TGF-β1 generated increased TGF-β1 levels at all time points. *P <0.05 (solid line) versus respective untreated controls for active TGF-β1 comparison. *P <0.05 (dotted lines) versus respective untreated controls for latent TGF-β1 comparison. Data are represented as mean ± SD, calculated from four independent studies in triplicate, and expressed as fold changes over respective controls. SD, standard deviation; TGF-β1 transforming growth factor-β1.

Focusing on single and double 4 h TGFβ1 pulses, cell layers were washed extensively with Hank’s balanced salt solution (HBSS) to remove recombinant TGF-β1. Substantial levels of endogenously produced active TGF-β1 levels were detectable in culture media 24 h post-pulse and had reverted to baseline at days 7 and 14 (Figure 3). In contrast, we observed an increased amount of latent TGF-β1 levels at all assessed time points as revealed when latent TGF-β1 in the samples were activated using hydrochloric acid to measure the total amount of TGF-β1 available.

### No apparent evidence for epigenetic modifications in selected fibrosis-related genes after TGF-β1 pulsing

Considering epigenetic changes as a basis for the long-lasting effects of TGF-β1 pulses, we studied DNA methylation events on the ACTA2 and COL1A1 genes after TGF-β1 pulse(s). We did not find significant DNA methylation modifications in our model (Table 2).

**Table 2:** DNA gene methylation levels remain unchanged after TGF-β1 pulses

**Amplicon**	**CpG site probed**	**4 h TGF-β1 pulse**			**2** × **4 h TGF-β1 pulses**		
		**Day 1**	**Day 7**	**Day 14**	**Day 1**	**Day 7**	**Day 14**
**ACTA2 (1)**	11	0.5 ± 0.5	0.85 ± 0.18	0.43 ± 0.33	0.86 ± 0.54	1.0 ± 0.41	1.75 ± 0.63
	12	1.0 ± 0.47	1.33 ± 0.63	0.6 ± 0.39	1.5 ± 1.06	0.67 ± 0.33	0.83 ± 0.23
	14	0.97 ± 0.18	1.26 ± 0.19	3.3 ± 2.26	1.58 ± 1.12	1.65 ± 0.36	0.7 ± 0.41
	16	0.81 ± 0.43	0.78 ± 0.50	0.93 ± 0.54	0.98 ± 0.29	1.78 ± 0.53	1.0 ± 0.36
	17	1.07 ± 0.32	1.08 ± 0.64	1.33 ± 0.63	1.2 ± 0.96	3.67 ± 2.92	0.5 ± 0.18
	20.21	1.0 ± 0.52	1.0 ± 0.1	0.71 ± 0.51	1.0 ± 0.51	1.0 ± 0.81	1.0 ± 0.67
	22	0.71 ± 0.25	1.4 ± 0.93	1.0 ± 0.71	0.43 ± 0.1	0.5 ± 0.33	1.0 ± 0.4
	23	0.71 ± 0.83	2.0 ± 1.54	2.0 ± 1.41	1.25 ± 1.06	1.76 ± 1.5	2.0 ± 1.18
	24	0.99 ± 0.63	1.2 ± 0.66	1.0 ± 0.29	0.71 ± 0.5	0.67 ± 0.31	2.0 ± 1.5
	25	1.0 ± 0.67	1.5 ± 1.23	1.0 ± 0.51	1.0 ± 0.3	1.0 ± 0.67	1.0 ± 0.67
**ACTA2 (2)**	1	4.0 ± 2.65	2.33 ± 1.19	1.0 ± 0.67	0.83 ± 0.24	1.75 ± 0.6	0.67 ± 0.31
	2	0.57 ± 0.11	1.67 ± 0.77	0.64 ± 0.15	1.14 ± 1.06	0.9 ± 0.78	0.56 ± 0.18
	4	1.31 ± 0.36	0.94 ± 0.08	0.57 ± 0.27	0.97 ± 0.41	1.15 ± 0.17	0.84 ± 0.25
	5	1.0 ± 0.67	2.0 ± 1.41	0.6 ± 0.32	0.33 ± 0.47	0.8 ± 0.23	1.0 ± 0.89
	10	0.99 ± −.67	0.83 ± 0.23	0.75 ± 0.35	1.75 ± 0.63	1.0 ± 0.47	1.0 ± 0.4
	11	2.0 ± 1.82	2.5 ± 0.71	0.33 ± 0.49	0.5 ± 0.39	1.0 ± 0.71	1.67 ± 0.92
	12.13	1.75 ± 0.36	0.4 ± 0.12	0.83 ± 0.46	1.0 ± 0.89	0.5 ± 0.18	1.0 ± 0.29
	14.15.16	0.69 ± 0.13	0.9 ± 0.14	0.81 ± 0.17	0.73 ± 0.45	0.78 ± 0.38	0.73 ± 0.35
**COL1A1 (1)**	3	1.0 ± 0.40	1.5 ± 0.5	1.33 ± 0.63	1.54 ± 0.43	1.0 ± 0.45	1.40 ± 0.39
	8.19	0.67 ± 0.56	0.60 ± 0.33	0.75 ± 0.53	0.92 ± 0.24	0.91 ± 0.28	0.73 ± 0.12
**COL1A1 (2)**	2	1.0 ± 0.81	1.33 ± 0.3	1.0 ± 0.47	1.67 ± 0.7	1.0 ± 0.71	0.83 ± 0.45
	7	1.4 ± 0.49	0.5 ± 0.33	1.75 ± 0.35	1.0 ± 0.71	1.5 ± 0.35	1.62 ± 0.60
	8.9	0.92 ± 0.59	2.0 ± 0.58	0.36 ± 0.41	1.33 ± 0.98	0.57 ± 0.38	1.4 ± 0.94
	11.12	0.67 ± 0.32	2.0 ± 1.8	1.0 ± 0.67	0.75 ± 0.35	0.75 ± 0.45	0.7 ± 0.51

ACTA2 and COL1A1 were not regulated by DNA methylation in response to TGF-β1 pulse(s). Changes in methylation levels >10% are considered significant. ACTA2 and COL1A1 genes expressed low methylation levels and no overt changes after TGF-β1 pulse(s). *ACTA1* actin alpha 1, *COL1A1* collagen, type I, alpha 1, *TGF-β1* transforming growth factor-β1.

### Myofibroblast generating ECM contained higher amounts of LTBP-1, collagen I and a higher degree of lysyl oxidase-mediated crosslinks

**Figure 4:**
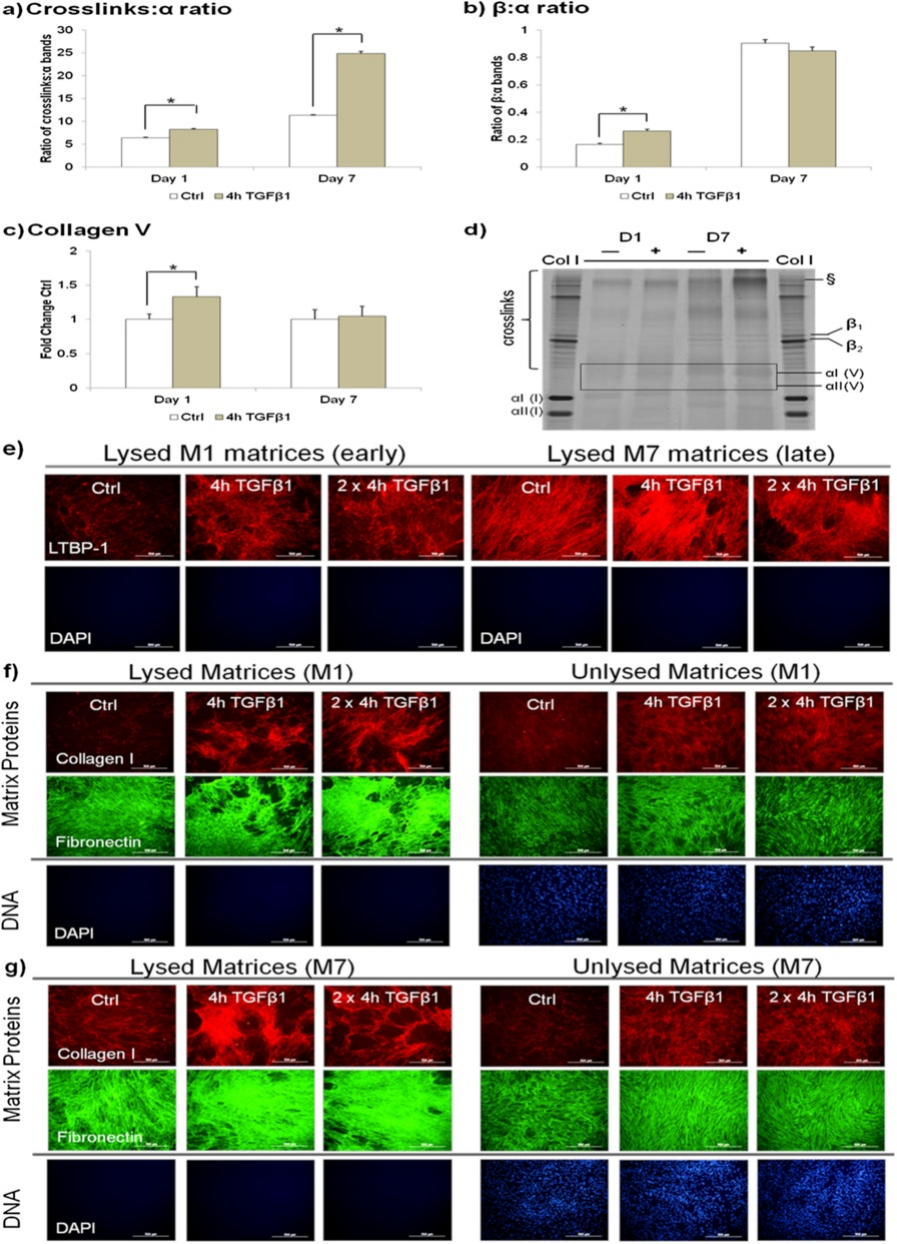
**TGF-β1 pulses increased matrix crosslinks, LTBP-1 and collagen I deposition.** Fibroblasts were pulsed with or without TGF-β1 for 4 h or 2 × 4 h. ECM was decellularised days 1 and 7 post-pulse. Normalised densitometric SDS-PAGE ratios of the **(a)** crosslinks:α; **(b)** β:α bands of the pepsin-digested ECM; **(c)** collagen V; and **(d)** corresponding silver-stained gel. The formation of high molecular weight aggregates of pepsin-resistant collagen is indicated by §; **(e)** LTBP-1 (red); **(f)** collagen I (red); **(g)** fibronectin (green) and nuclei stained with DAPI (blue) of the TGF-β1-pulsed ECM. Scale bars = 200 μM. *P <0.05 versus respective untreated controls. Data are represented as mean ± SD, calculated from three independent studies in triplicate, and expressed as fold changes over respective controls. ECM, extracellular matrix; LTBP-1, latent transforming growth factor binding protein-1; SD, standard deviation; TGF-β1 transforming growth factor-β1.

We proceeded to assess the ECM components of the TGF-β1-pulsed ECM. We observed increased matrix crosslinks on the M1 and M7 ECM. Also, increased β:α crosslinking ratio, collagen V, collagen I deposition and latent transforming growth factor binding protein-1 (LTBP-1) expression on the ECM decellularised 1 day post-pulse was observed (Figure 4). M7 matrices showed particularly under TGF-β1 pulses an apparent normalisation of the β:α ratio, however, high molecular weight bands in the γ region and above, and close to the slot become prominent, indicating the formation of high molecular weight aggregates of pepsin-resistant collagen (Figure 4d).

### ECM generated under TGF-β1 pulses induced myofibroblast phenotype while normal ECM downmodulated it

**Figure 5:**
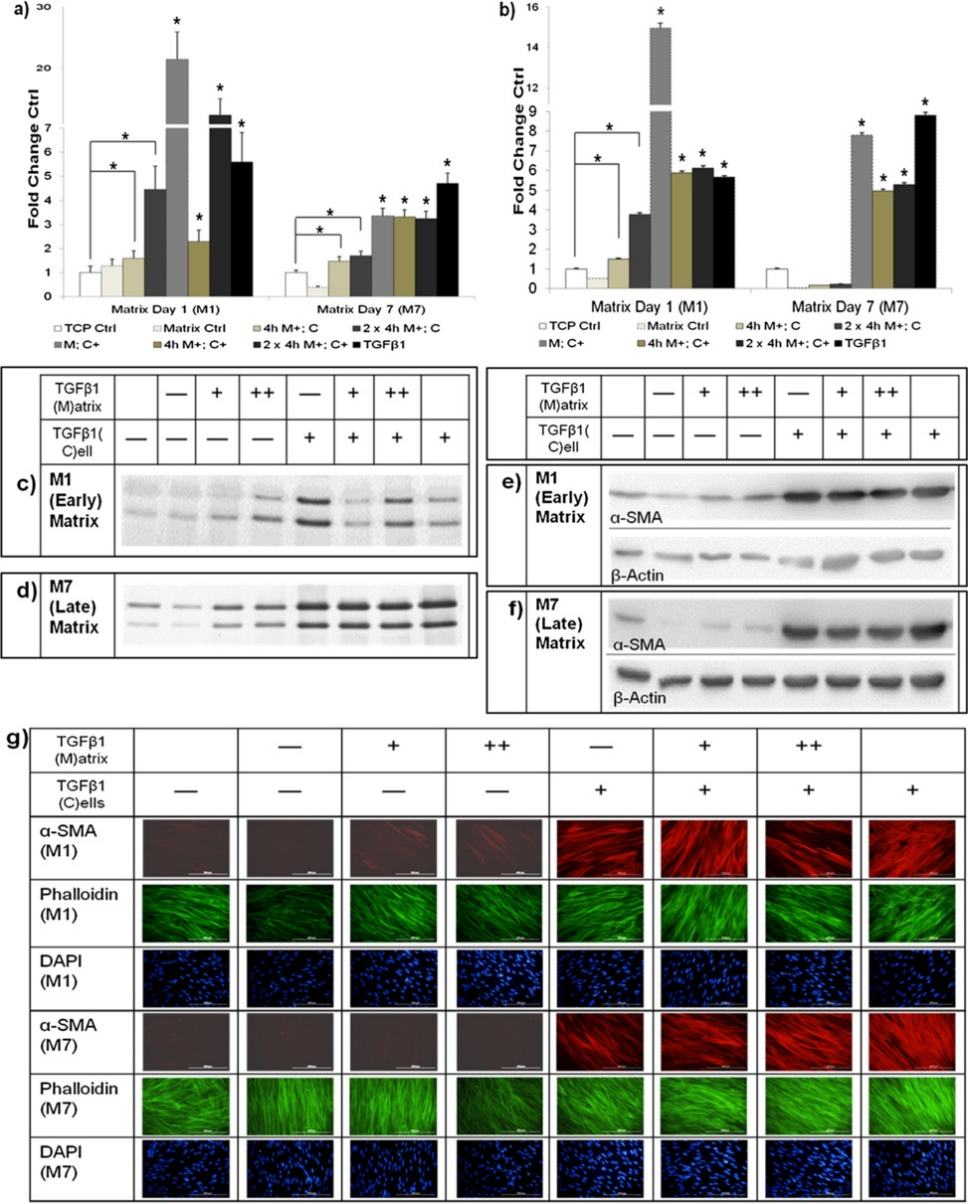
**TGF-β1-pulsed ECM influenced the myofibroblast phenotype.** Untreated fibroblasts were seeded onto decellularised TGF-β1-pulsed ECM. Classic myofibroblast markers were assessed 7 days post-culture. **(a)** Normalised densitometric SDS-PAGE analysis of the 24 h collagen secretion rate; **(b)** densitometric analysis of α-SMA normalised to β-actin expression; representative **(c-f)** corresponding SDS-PAGE gels and immunoblots of M1 and M7 ECM; **(g)** immunofluorescence images showing presence and distribution of α-SMA (red); F-actin (phalloidin, green) and nuclei stained with DAPI (blue). TCP control, matrix control and fibroblasts reseeded onto 4 h and 2 × 4 h TGF-β1-pulsed ECM images are modified to highlight α-SMA expression. Scale bars = 200 μM. *P <0.05 versus respective untreated controls. Data are represented as mean ± SD, calculated from three independent studies in triplicate, and expressed as fold changes over respective TCP controls. TGF-β1 ECM: ‘+’ denotes a 4 h and ‘++’ 2 × 4 h TGF-β1 pulse(s) on decellularised ECM. TGF-β1 cells: ‘+’ denotes a 24 h TGF-β1 pulse on reseeded fibroblasts. α-SMA, α-smooth muscle actin; ECM, extracellular matrix; SD, standard deviation; TCP, tissue culture plastic; TGF-β1 transforming growth factor-β1.

**Figure 6:**
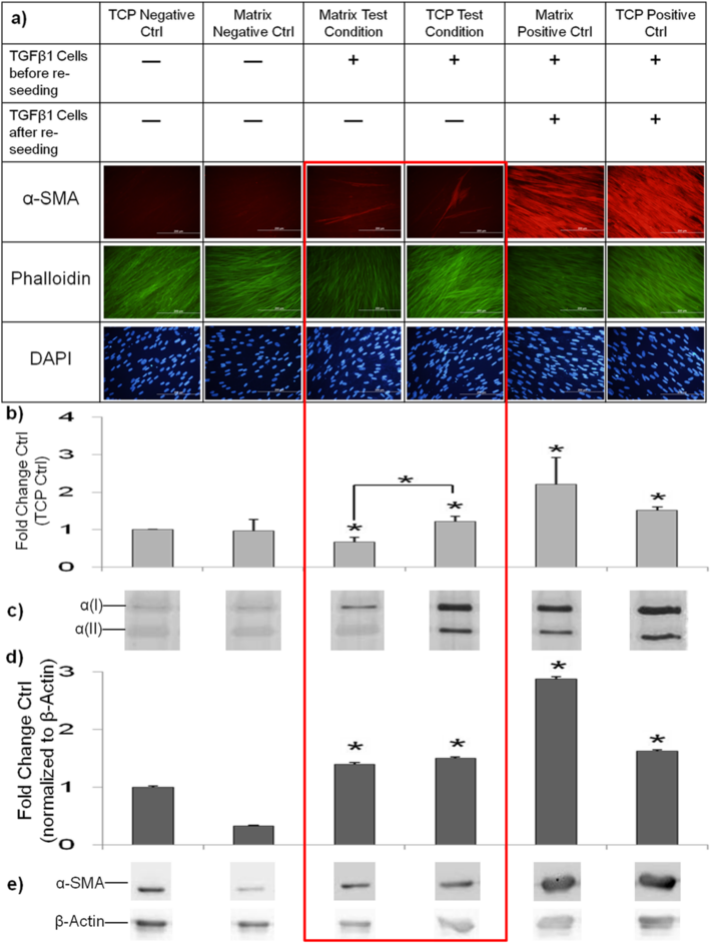
**Fibroblast ECM downmodulated collagen I production in myofibroblasts.** Four-day TGF-β1-treated myofibroblasts were dispase passaged and reseeded onto fibroblast ECM. Classic myofibroblast markers were assessed 7 days post-culture. **(a)** Immunofluorescence images showing the presence and distribution of α-SMA (red); F-actin (phalloidin, green) and nuclei stained with DAPI (blue). Scale bars = 200 μM. **(b)** Normalised densitometric SDS-PAGE analysis of the 24 h collagen secretion rate; **(c)** corresponding silver-stained gel; **(d)** densitometric analysis of α-SMA immunoblots normalised to β-actin bands; and **(e)** corresponding immunoblots. *P <0.05 versus respective to TCP controls. *P <0.05 versus respective to myofibroblasts passaged and reseeded on TCP. Data are represented as mean ± SD, calculated from duplicate studies in triplicate, and expressed as fold changes over respective controls. α-SMA, α-smooth muscle actin; ECM, extracellular matrix; SD, standard deviation; TCP, tissue culture plastic; TGF-β1 transforming growth factor-β1.

To investigate the phenotypic influence of ECM-mediated (produced after TGF-β1 pulses) myofibroblast induction, fibroblasts were pulsed with or without TGF-β1 for 4 h and 2 × 4 h. Fibroblasts were then removed by detergent treatment and resulting decellularised ECM (Additional file 4: Figure S4) was reseeded with previously untreated fibroblasts. We observed that in particular, doubly TGF-β1-pulsed ECM was able to induce a myofibroblastic phenotype (Figure 5). Furthermore, the myofibroblast-inducing properties were strongest in ECM decellularised 1 day post-pulse and were slightly diminished (only α-SMA expression increased) in ECM 7 days after decellularisation. *Vice versa*, we seeded myofibroblasts onto ECM from non-TGF-β1-treated fibroblasts. To increase the yield of ECM deposition as according to [19], matrix formation was performed in the presence of macromolecular crowding. To generate myofibroblasts, we employed 4 days of TGF-β1 treatment (the current ‘gold standard’ in the field). As we noted a loss of phenotype using conventional trypsin passaging (Additional file 5: Figure S5), we replaced this method by using dispase, which preserved the phenotype (Additional file 6: Figure S6). We observed that myofibroblasts cultured on fibroblast ECM normalised their collagen I production (Figure 6 and Additional file 7: Figure S7).

## Discussion

A myriad of short-lived signals permeate living tissues in health and disease. In fibrosis, TGF-β-mediated processes play an important role in the polarisation of mesenchymal cells into myofibroblasts. In this study, we considered the effects of pulsed TGF-β1 on WI-38 cells and made two significant observations. First, that a short TGF-β1 pulse has a surprisingly long-lasting effect as evidenced by the generation of a *bona fide* myofibroblast phenotype. This suggests some phenotypic signal retention mechanism or information storage in or around the cell. Second, this effect is transient; it wears off. This suggests dampening of a phenotype-maintaining signal and erasure of stored information. To start with, pulsatile impact of GF on target cells is not well studied, but it is conceivable that it offers advantages over continuous exposure. It avoids downregulation of receptors on target cells as a possible response to signal bombardment [16]. While this keeps cells continuously responsive, it also reduces inertia in a feedback system. In contrast to hormones, GF work locally in a diffusion perimeter around releasing cells and can be stored and moved around in the ECM [20]. Release of TGF-β1 can be due to direct secretion (for example by inflammatory) cells or by proteolytic or mechanical release from matrix storage points, but it is not known whether these modes generate a continuous or a pulsed/intermittent scenario. Modelling the latter, we found a single TGF-β1 pulse of 4 h or even 30 min to be sufficient to induce and maintain a myofibroblast phenotype for up to 1 week; an additional pulse 1 day later doubled the duration of the effect. This was similar to the duration of effects obtained with continuous 4 days of TGF-β1 treatment, a classical *in vitro* treatment scheme in experimental biology. Twenty-four hours after a single TGF-β1 pulse, fibrosis-related genes were maximally upregulated, while α-SMA and collagen I secretion levels took a further 6 days to peak. We therefore propose four levels of signal propagation and message maintenance to explain the transience of the TGF-β1-induced myofibroblast phenotype. First, computational modelling as well as experimental work has shown that TGF-β1 pulses as short as 30 s can induce a SMAD cascade, with elevated phosphorylation levels lasting for 4 h [21]. However, long-term observations were not made in this publication and classically, the SMAD activation cascade wears off in a matter of 3 to 4 h [22]. Therefore, SMAD phosphorylation would not plausibly serve as the maintainer of a myofibroblast phenotype for the observed time period. However, we noticed with interest that a single pulse of exogenous TGF-β1 leads to subsequent release of endogenous active TGF-β1 and, therefore, to an autocrine stimulation that is dampened during the first 24 h. Further work needs to be done to determine the pulsatile nature of this autocrine TGF-β1 release and resultant SMAD activation period. We would currently speculate that the first pulse does not downregulate TGF-β1 receptors, but the following autocrine self-stimulation might, thus leading to the first dampening of signal.

At the next level of intracellular changes that could transiently stabilize the myofibroblast phenotype after a single TGF-β1 pulse, we considered epigenetic modifications affecting DNA methylation. We could not establish significant CpG methylation changes for selected regions of selected TGF-β1-responsive genes (ACTA2, COL1A1). Also, no changes in histone H3 methylation were seen in the lysyl hydroxylase PLOD2 gene [Ruud Bank, University of Eindhoven, personal communication, 2012]. This does not rule out methylation changes in other genes and regions under the influence of TGF-β1. For example, the CpG island promoter of RASAL1, a member of the RAS-GAP family was found to be consistently hypermethylated in fibrotic renal fibroblasts correlating with suppressed expression levels. However, this region was never found to be methylated in acute kidney injury, which is associated with transient fibroblast activation and transient suppression of RASAL1 transcription. It is assumed that hypermethylation serves as a mechanism to imprint this pathway, preventing the fibrotic fibroblasts from returning to their quiescent state [23]. Such an imprint pathway might be active in systemic scleroderma fibroblasts [24,25] of which a subset shows a constitutive activation of the (TGF)β/Alk1/SMAD1 signalling pathway. As in our model, the observed transitory character of fibroblast activation after a TGF-β1 pulse did not suggest that such an imprint mechanism is in operation. We considered as the next option, microenvironmental changes that could positively reinforce a myofibroblastic phenotype based on the notion of dynamic cell matrix reciprocity [26,27]. This term entails cellular influence on the matrix via matrix synthesis, degradation and remodelling [28,29], and in turn an ECM influence on cell decisions. We observed in matrices laid down by TGF-β-pulsed fibroblasts a markedly increased deposition of collagen I, collagen V and LTBP-1. LTBP-1 holds latent TGF-β1 in storage and is crosslinked to matrix proteins such as fibronectin [30], fibrillin-1 [31] and collagen I [32]. This suggests that latent TGF-β1 is increasingly stored in the nascent and later maturing matrix from which the active form can be released to support the phenotype. This observation would suffice to explain how a single pulse can maintain a myofibroblast signal after the initial autocrine TGF-β1 secretion has subsided, roughly after 24 h. A second pulse 24 h later would prolong the total TGF-β1 exposure time to at least 48 h (exogen plus endogen TGF-β1) and in this period more matrix can be deposited. Its major components, collagen I [33] and fibronectin FN ED-A domain [34] were already shown to induce the myofibroblast phenotype. In addition, myofibroblast contraction activates latent endogenous TGF-β1 stores on the ECM [35], thereby plausibly contributing to myofibroblast maintenance. We also observed increased collagen V deposition in TGF-β1-pulsed matrices. Increased deposition of collagen V and the subsequent thickening of lamina reticularis are implicated in subepithelial asthma fibrosis [36]. Collagen V co-assembles with collagen I to form heterotypic fibrils [37], its increased deposition mirrors that of collagen I. Collagen V is known to be highly expressed during tissue development, wound repair, and fibrogenesis. The collagen V propeptide PVCP-1230 was recently suggested as a biomarker for tissue remodelling in liver fibrosis [38].

Support stiffness is also increasingly discussed as a modulator of cellular phenotype. In this regard, the mechanical properties of ECM under TGF-β1 pulses could be altered through the activation of matrix crosslinking enzymes such as transglutaminase [39] and lysyl oxidase (LOX) [40]. We found biochemical evidence for an increased LOX-mediated collagen I crosslinking in the M1 and M7 matrices. A direct relationship between LOX crosslinking and matrix stiffness has recently been demonstrated for the pathological microenvironment of colorectal cancer in liver tissue [41]. Indeed, a stiff ECM has been also described to drive myofibroblast formation [42-45]. A new twist has been added recently to this apparent straightforward relationship with the discovery that an admixture of collagen V softens collagen I lattices [46]. More work needs to be done to determine whether an increased collagen V deposition might represent compensation mechanisms to control matrix stiffness.

We employed decellularised ECM that had been generated under TGF-β1 pulses and showed that it can induce and transiently maintain a myofibroblast phenotype in fibroblasts that have not been exposed to this GF. Maximal inductive capacity was present in M1 matrices (24 h after pulse). Considering the huge matrix losses during decellularisation (70 to 90%) the full efficacy of entirely intact matrices can be easily appreciated. The fading of the myofibroblast-inducing effect of M7 matrices coincided with a high degree of crosslinking and collagen V deposition. This suggests erasure of biochemical inductive signals due to dynamic turnover as effected by proteolytic remodelling of ECM. In a crossover experiment, we showed that matrix laid down by control (non-treated cells) normalised the collagen output of myofibroblasts. These observations tie in with previous findings that cultivation on matrigel [47] and cryopreserved amniotic membrane stromal surfaces [33] reversed the α-SMA expressing myofibroblast phenotype. α-SMA, however, was not normalised by the normal fibroblast matrices in our hands. More work needs to be done to study the role of proteolytic remodelling in erasing fibrogenic ECM signals, but it is tempting to speculate that the induction of matrix metalloproteinase activity in fibrotic tissue by hepatocyte growth factor reviewed in reference [48] in physiology and with therapeutic intent, might serve to wipe the slate clean and to interrupt a feed-forward loop that otherwise would lead to a chronic fibrotic state.

## Conclusions

Here, we have first demonstrated that short TGF-β1 pulse(s) exerts a long-lasting effect on normal fibroblasts with regard to attaining a myofibroblastic phenotype. This effect is based on cell-matrix reciprocity: the microenvironment appears to retain a memory of TGF-β1 insults in the form of biochemical cues, including a higher content of endogenous latent TGF-β1 that is produced as a response to a short exogenous active TGF-β1 pulse. On first sight, this would constitute a positive feed-back loop that should lead to perpetuation of the myofibroblast phenotype after a limited TGF-β1 impact. Surprisingly, this is not the case, a dampening effect ensues that we have to attribute to spontaneous remodelling of the matrix by the cells and the subsequent removal of myofibroblastic cues over time. Therefore, our model appears to emulate a typical tissue injury response in its transience. As limited as this *in vitro* system is, it illustrates, that it would take one 30 min TGF-β1 pulse every week only to maintain the myofibroblast phenotype. This has a bearing for the development of antifibrotic drugs and points to future research avenues to study fibrotic processes.

## Methods

### Cell culture and treatment of lung fibroblast line

Human fetal lung fibroblasts WI-38 (CCL-75; ATCC, Manassas, VA, USA) were cultured in 10% fetal bovine serum (FBS) Dulbecco’s modified Eagle’s medium (DMEM) (Gibco Invitrogen, Grand Island, NY, USA) in 5% CO2 at 37°C. The cells were cultured at low passage (passage number 6 to 8). Fibroblasts were seeded at 5 × 10^4^/well in 24-well plates or 1 × 10^5^/well in 12-well plates in 10% FBS DMEM. Cells were seeded at 70% and 50% of the above mentioned density for 7 and 14 days respectively. After 24 h to allow for cell attachment in 10% FBS, the fibroblasts were growth-arrested by DMEM media for 24 h. Subsequently, they were treated with or without 5 ng/ml of TGF-β1 pulses (R&D Systems, Minneapolis, MN, USA) in serum-free DMEM and 30 μg/ml L-ascorbic acid phosphate magnesium salt η-hydrate (ACA; Wako, Osaka, Japan), at the pulsed time points of 0.5 h and 4 h. Thereafter, TGF-β1 treatment was removed and cultures washed twice with HBSS. Cultures were maintained in 0.5% FBS DMEM and 30 μg/ml ACA, with medium changes every 4 days. Serum-free DMEM was used to assess the collagen content in culture media of the indicated follow-up day, where the culture media was changed and harvested after 24 h to analyse the collagen content.

### Biochemical analysis of collagen production

Harvested serum-free DMEM in the last 24 h of the culture period was harvested and digested with 25 μg/ml porcine gastrin mucosa pepsin (Roche, Basel, Switzerland). Collagen I deposition on the ECM was digested *in situ* with 250 μg/ml porcine gastric muscosa pepsin. Extracts were digested in 0.1 N HCl for 2 h and neutralized with 1 N NaOH. Extracts were then visualized under non-reducing conditions using 5% resolving/3% stacking SDS-PAGE gel electrophoresis as outlined in [49]. Protein bands were stained with the SilverQuest™ kit according to manufacturer’s protocol (Invitrogen, Carlsbad, CA, USA). Densitometric analysis of wet gels was performed on the collagen α1(I)-bands with the GS-800™ calibrated densitometer and analyzed by the Quantity One v4.5.2 image analysis software (Bio-Rad, Hercules, CA, USA).

### Adherent cytometry

For the normalisation of collagen I secretion rate, fibroblasts were stained with 4′,6-diamidino-2-phenylindole (DAPI); Molecular Probes, Eugene, OR, USA) after absolute methanol fixation at days 1, 7 and 14. Nine image sites covering 71% of the total well area were acquired at 2× magnification using a Nikon TE600 fluorescence microscope (Nikon Corp., Tokyo, Japan) an automated Ludl stage (BioPrecision 2; Ludl Electronic Products Ltd., Hawthorne, NY, USA) and analysed using the Metamorph™ Imaging System software (Molecular Devices, Downingtown, PA, USA) as described in [50]. A nucleus was defined as a fluorescent region with a length of 10 to 20 μM and pixel intensity value of 10 units above background.

### Immunoblotting

Western blots were performed according to [51]. Briefly, proteins were extracted from the cell layer with loading buffer comprising of 50 mM Tris-HCl pH 6.8, 2% SDS, 0.1% bromophenol blue and 10% glycerol and protease inhibitor cocktail (Roche, Basel, Switzerland). Extracts were separated under non-reducing conditions on 12% resolving/3% stacking SDS-PAGE gel electrophoresis with 5 mM DTT. Proteins were electroblotted onto a nitrocellulose membrane. Immunodetection was carried out in Tris-buffered saline Tween-20 at pH7.6 (50 mM Tris-base, 150 mM NaCl and 0.05% Tween 20). Membrane was blocked with 5% non-fat milk for 1 h. Primary antibodies against α-SMA (1:500) and β-actin (1:1000) were from mouse (Sigma-Aldrich, St. Louis, MO, USA). Primary antibody incubation was carried out for 1.5 h. Membrane was incubated for 1 h with secondary antibody goat anti-mouse HRP (1:3000, Dako, Glostrup, Denmark). Membrane was washed with buffer three times after antibody incubation. Blots were developed with the Pierce Western blotting detection system (Thermo Scientific Pierce, Rockford, IL, USA) for 3 min. Chemiluminescence signal was captured with the VersaDoc Imaging System model 5000 and analysed with the Quantity One v4.5.2 image analysis software (Bio-Rad, Hercules, CA, USA).

### Immunocytochemistry

Cell layers were washed with HBSS and fixed with methanol-free 3.7% formaldehyde (Thermo Scientific Pierce, Rockford, IL, USA) at room temperature (RT) for 10 min. The cell membrane was permeabilised with 0.1% Triton X-100 for 3 min. After washes with PBS, non-specific sites were blocked with 3% BSA for 1 h followed by incubation with primary antibody, α-SMA (1:100; Dako, Glostrup, Denmark), collagen I (1:1000; Sigma-Aldrich, St. Louis, MO, USA), rabbit anti-fibronectin (1:100; Dako, Glostrup, Denmark), or rabbit anti-LTBP-1 (1:200, a gift from Dr. Carl-Henrik Heldin, Helsinki, Finland) for 1.5 h. Secondary antibodies were goat anti-mouse AlexaFluor594, chicken anti-rabbit AF488, goat anti-rabbit AlexaFluor594 (1:400; Molecular Probes, Eugene, OR, USA) and AlexaFluor488 phalloidin (1:100; Molecular Probes, Eugene, OR, USA). Cell nuclei were counterstained with DAPI. Images were acquired with an Olympus LX71 epifluorescence microscope (Olympus, Tokyo, Japan). All digital images were background-subtracted based on conjugate control.

### Quantitative reverse transcriptase real-time polymerase chain reaction (RT-PCR)

Total RNA was isolated from cell extracts using Trizol™ reagent (Invitrogen, Carlsbad, CA, USA) and the RNeasy mini kit (Qiagen, Valencia, CA, USA). RNA concentration was determined using NanoDrop (NanoDrop Technologies, Wilmington, DE, USA). A total of 100 ng of total RNA was reverse-transcribed using the SuperScript III reverse transcriptase (Invitrogen, Carlsbad, CA, USA) with oligo(dT) primers according to the manufacturer’s protocol. Real-time polymerase chain reaction was carried out using 2 μL of cDNA, 10 μL of Maxima™ SYBR Green/ROX qPCR Master Mix (Thermo Fisher Scientific, Waltham, MA, USA) and 0.3 μM of primers in a reaction volume of 20 μL. All reactions were performed on the real-time Mx3000P (Stratagene, La Jolla, CA, USA). The thermal cycling program for all polymerase chain reactions was: 95°C for 15 min, followed by 40 cycles of amplifications, consisting of a denaturation step at 94°C for 15 s, an annealing step at 55°C for 30 s, and an extension step at 72°C for 30 s. Fibrogenic genes analysed were ACTA2, FZD8, NOX4 and TSPAN2. Primers were designed using the Oligo6.0 program (National Biosciences Inc., Plymouth, MN, USA) and are listed in Table 3. The level of expression of the target genes, normalised to glyceraldehyde 3-phosphate dehydrogenase (GAPDH) was calculated using the ΔΔCT formula and expressed as fold-change controls.

**Table 3:** Primer sequences of selected fibrogenic genes

**Gene**	**Primer sequence**
**GAPDH**	Forward primer: 5'- GTCCACTGGCGTCTTCACCA -3'
	Reverse primer: 5'- GTGGCAGTGATGGCATGGAC -3'
**ACTA2**	Forward primer: 5'- TTCAATGTCCCAGCCATGTA-3'
	Reverse primer: 5'- GAAGGAATAGCCACGCTCAG-3'
**FZD8**	Forward primer: 5'- AGACAGGCCAGATCGCTAACT-3'
	Reverse primer: 5'- AAGCGCTCCATGTCGATAAG-3'
**NOX4**	Forward primer: 5'- GGCCAGAGTATCACTACCTCC-3'
	Reverse primer: 5'- GTTCGGCACATGGGTAAA-3'
**TSPAN2**	Forward primer: 5'- TTCATGTGTGATCTGCGTGTT-3'
	Reverse primer: 5'- TGGGAGCGAAATAGGTTGT-3'

Primers were designed using the Oligo6.0 bioinformatics program, and analysed using quantitative real-time polymerase chain reaction (RT-PCR). *ACTA2* actin alpha 2, *FZD8* frizzled family receptor 8, *GAPDH* glyceraldehyde 3-phosphate dehydrogenase, *NOX4* NADPH oxidase 4, *TSPAN2* transmembrane glycoprotein tetraspanin 2.

### TGF-β1 Quantikine enzyme-linked immunosobent assay (ELISA)

The expression of TGF-β1 was determined with commercially available ELISA kit (Human TGF-β1 Quantikine ELISA Kit; R&D Systems, Minneapolis, MN, USA). At the indicated endpoint, culture media was changed and harvested after 24 h to analyse TGF-β1 content supernatant by sandwich ELISA according to specialized procedures described in the manufacturer’s protocol.

### MassARRAY: DNA extraction, bisulfite-conversion - PCR and Spot-fire

Genomic DNA was isolated from cell extracts using the DNeasy mini kit (Qiagen, Valencia, CA, USA), and concentration determined using NanoDrop (NanoDrop Technologies, Wilmington, DE, USA). DNA methylation was measured with the Sequenom MassARRAY Compact System (Sequenom, San Diego, CA, USA) [52]. Briefly, gene-specific amplification of bisulfite-treated DNA was followed by *in vitro* transcription and analysis by matrix-assisted laser desorption ionization time-of-flight (MALDI-TOF) mass spectrometry. Sequenom assay design and methods were according to procedures outlined in the manufacturer’s protocol. 1 μg DNA was bisulfite-converted using EZ DNA Methylation kit (Zymo Research, Irvine, CA, USA). PCR primers specific (Table 4) for bisulfite-converted DNA were designed using the UCSC Genome Browser [53] and Methprimer [54]. Each of the reverse primer’s contained a T7-promoter tag for *in vitro* transcription (5′-cagtaatacgactcactatagggagaaggct-3′), and the forward primer was tagged with a 10mer to balance Tm (5′-aggaagagag-3′). Bisulfite-treated DNA was PCR-amplified using HotStar Taq Polymerase (Qiagen, Valencia, CA, USA) in 5 μL reactions and treated with shrimp alkaline phosphatase (Sequenom, San Diego, CA, USA) for 20 min at 37°C and then at 85°C for 5 min. *In vitro* transcription/uracil-cleavage reaction was carried out in 7 μL reactions using Sequenom T-cleavage reagent mix. Transcription cleavage products were desalted with 6 mg of CLEAN-Resin and 20 nL spotted on a 384-pad SpectroCHIP (Sequenom, San Diego, CA, USA) using a MassARRAY nanodispenser (Samsung, Seoul, South Korea). Mass spectra were acquired using a MassARRAY MALDI-TOF MS (Bruker-Sequenom, San Diego, CA, USA) and peak detection, signal-to-noise calculations and quantitative CpG-site methylation performed using proprietary EpiTyper software v1.0 (Sequenom, San Diego, CA, USA). Samples that failed to give reliable PCR product or produced spectra with low confidence levels (<2.9 in EpiTyper) were excluded from analysis. For fragments containing a single CpG site, DNA methylation was calculated by the ratio of methylated to unmethylated fragments. Lower boundary limitations imposed by Sequenom analysis treat cleavage products containing multiple CpG sites as single units and methylation values reported were weighted averages across the unit (referred to as a CpG group). DNA quality and no-template controls, 0% and 100% methylated DNA were included in all assays.

**Table 4:** Amplicons, genomic coordinates and primer sequences of the extended promoter regions measured

**Amplicon**	**Genomic coordinates**	**Primer sequence**		**CpG**
**ACTA2 (1)**	chr10:90,750,187-90,750,540	Fwd	5'-TAGTTAGGGTTGGTTTTAGGGTGT-3'	19
		Rev	5'-CCTAAAATAAACATACCAACCACTACA-3'	
**ACTA2 (2)**	chr10:90,750,828-90,751,169	Fwd	5'-TTTGTTTTGAAGGTTGTAGGTTTTTT-3'	26
		Rev	5'-ACTATTAAAACCTTCCCTCAAACCC-3'	
**COL1A1 (1)**	chr17:48,278,603-48,278,899	Fwd	5'-AGTTTATATGTTTAGGGTTTAGATATGTT-3'	19
		Rev	5'-CCAAAATAAACTCCCTCCTATCTCA-3'	
**COL1A1 (2)**	chr17:48,278,860-48,279,232	Fwd	5'-AGTATTTTTGGTTTAGGTTGGG-3'	17
		Rev	5'-CACAAAACTAAACATATCTAAACCCT-3'	

PCR-specific primers were designed using the UCSC Genome Browser and CpG sites predicted using the Methprimer algorithm. *ACTA2* actin alpha 2, *COL1A1* collagen, type I, alpha 1.

### Generation and decellularisation of TGF-β1-pulsed and untreated fibroblast ECM

To generate TGF-β1-pulsed ECM, growth-arrested fibroblasts were pulsed with or without TGF-β1 and maintained for either 1 day post-pulse (early matrix; denoted as M1) and 7 days post-pulse (late matrix, denoted as M7); and to generate fibroblast ECM, growth-arrested untreated fibroblasts were supplemented with 0.5% FBS DMEM and crowder cocktail made up of 37.5 mg/ml Ficoll (Fc) 70 and 25 mg/ml Ficoll 400. At the indicated analysis time point, untreated fibroblast and TGF-β1-pulsed monolayers were washed twice with HBSS then treated with 0.5% DOC (Prodotti Chimici E Alimentari, S.P.A 2003030085), supplemented with 0.5× protease inhibitor cocktail in water for 15 min on ice four times, followed by 0.5% DOC in phosphate-buffered saline (PBS) on ice two times. ECM were washed with PBS thrice, and then treated with 0.5 mg/ml DNAse (US Biological, Swampscott, MA, USA) at 37°C for 1 h. To remove residual detergent and DNAse activity, ECM was washed with PBS three times before fibroblasts or myofibroblasts were seeded onto the ECM. ECM were analysed by fixing with absolute methanol for 10 min, air drying for the next 30 min, followed by immunocytochemistry. At the indicated endpoint, the 24 h collagen I content and α-SMA expression was assessed.

### Statistical analysis

Statistical analysis was performed using GraphPad software (GraphPad Software Inc., San Diego, CA, USA). The statistical significance between groups was determined using the Student’s *t*-test, two-tailed distribution with unequal variance. Probability values of *P* <0.05 (95% confidence interval) in comparison with controls were accepted as the level of statistical significance.

### Approvals

All experimental work in this publication involves commercially available human cell lines only was conducted after expedited review with the approval of the Institutional Review Board of the National University of Singapore (IRB reference code 09-449E).

## Competing interests

The authors declare that they have no competing interests.

## Authors' contributions

AT conceived, designed, analysed and participated in its design and coordination all the experiments outlined in the manuscript. SK participated in the experimentation of assessing the effects of the fibroblast extracellular matrix on the myofibroblast phenotype. The DNA methylation experiments were designed, analysed and conducted by LC and AS. AT and MR wrote the paper. All authors read and approved the final manuscript.

## Supplementary Material

Additional file 1: Figure S1 SDS-PAGE gels comparing short TGF-β1 pulse(s) and 4 days of continuous TGF-β1 treatment. (a) Growth-arrested fibroblasts were treated with or without TGF-β1 according to the cell culture setup comparing single and double TGF-β1 pulses with the traditional 4 days of TGF-β1 treatment. Corresponding silver-stained SDS-PAGE gels for the (b) 0.5 h, (c) 4 h; (d) 2 × 0.5 h, (e) 2 × 4 h; and (f) 4 days of TGF-β1 treatments from which densitometric analysis of the 24 h collagen secretion rate was derived. TGF-β1 transforming growth factor-β1. Click here for file

Additional file 2: Figure S2 Immunoblots comparing short TGF-β1 pulse(s) and 4 days of continuous TGF-β1 treatment. (a) Growth-arrested fibroblasts were treated with or without TGF-β1 according to the cell culture setup comparing single and double TGF-β1 pulses with the traditional 4 days of TGF-β1 treatment. Corresponding α-SMA immunoblots for the (b) 0.5 h, (c) 4 h; (d) 2 × 0.5 h, (e) 2 × 4 h; and (f) 4 days of TGF-β1 treatments from which densitometric analysis of α-SMA normalised to β-actin bands was derived. α-SMA, α-smooth muscle actin; TGF-β1 transforming growth factor-β1. Click here for file

Additional file 3: Figure S3 Immunofluorescence images comparing short TGF-β1 pulse(s) and 4 days of continuous TGF-β1 treatment. Immunofluorescence images showing presence and distribution α-SMA (red); F-Actin (phalloidin, green) and nuclei stained with DAPI (blue) from (a) 4 h; (b) 2 × 4 h TGF-β1 pulsed; and (c) 4 days of TGF-β1-treated cell layers. Scale bars = 200 μM. *P < 0.05 versus respective untreated controls. α-SMA, α-smooth muscle actin; TGF-β1 transforming growth factor-β1. Click here for file

Additional file 4: Figure S4 TGF-β1-pulsed decellularised ECM was free from cellular and matrix residues. The absence of actin and DNA residues was observed in lysed ECM. Representative immunoblots of (a) 4 h; and (b) 2 × 4 h TGF-β1-pulsed ECM. Representative ICC pictures of (e) early M1; and (f) late M7 ECM showing the presence and distribution of cytoskeletal element F-actin (phalloidin, green); and nuclei stained with DAPI (blue). ‘L’ denotes decellularised ECM and ‘UL’ the unlysed ECM (positive matrix control). ECM, extracellular matrix; TGF-β1 transforming growth factor-β1. Click here for file

Additional file 5: Figure S5 Trypsin-EDTA attenuated the myofibroblast phenotype. (a) Cell culture setup of TGF-β1-treated myofibroblasts and subsequent sub-culture. Fibroblasts were treated with and without TGF-β1 for 4 days before passaging using trypsin. Myofibroblasts were replated onto TCP. (b) Normalised densitometric SDS-PAGE analysis of the 24 h collagen secretion rate by induced fibroblasts; (c) corresponding silver-stained gel; (d) densitometric analysis of α-SMA immunoblots normalised to β-actin bands; (e) corresponding immunoblot; and (f) immunofluorescence images showing presence and distribution α-SMA (red); F-Actin (phalloidin, green) and nuclei stained with DAPI (blue) from passaged myofibroblasts. Scale bars = 200 μM. *P <0.05 versus respective untreated controls. Data are represented as mean ± SD, calculated from three independent studies in triplicate, and expressed as fold changes over respective controls. α-SMA, α-smooth muscle actin; SD, standard deviation; TCP, tissue culture plastic; TGF-β1 transforming growth factor-β1. Click here for file

Additional file 6: Figure S6 Dispase passaging of myofibroblasts preserved phenotype but reduced α-SMA expression. Fibroblasts were treated with or without TGF-β1 for 4 days. Thereafter, dispase was employed to passage myofibroblasts and cultures maintained for further 7 days post-replating on TCP. Immunofluoresence images showing the presence and distribution of α-SMA (red); and nuclei stained with DAPI (blue). Scale bars = 500 μM. α-SMA, α-smooth muscle actin; TCP, tissue culture plastic; TGF-β1 transforming growth factor-β1. Click here for file

Additional file 7: Figure S7 Immunoblots from cell layers of myofibroblasts reseeded on fibroblast ECM. Corresponding consolidated immunoblot showing the persistence of α-SMA expression after 4-day TGF-β1-treated myofibroblasts were dispase passaged and reseeded onto fibroblast ECM. ECM, extracellular matrix; TGF-β1 transforming growth factor-β1. Click here for file
