# Supplementary figures and images for: Cellular re- and de-programming by microenvironmental memory: why short TGF-β1 pulses can have long effects
Source: Fibrogenesis Tissue Repair. 2013 Jun 19;6:12. doi: 10.1186/1755-1536-6-12 (PMC3702516; doi:10.1186/1755-1536-6-12)

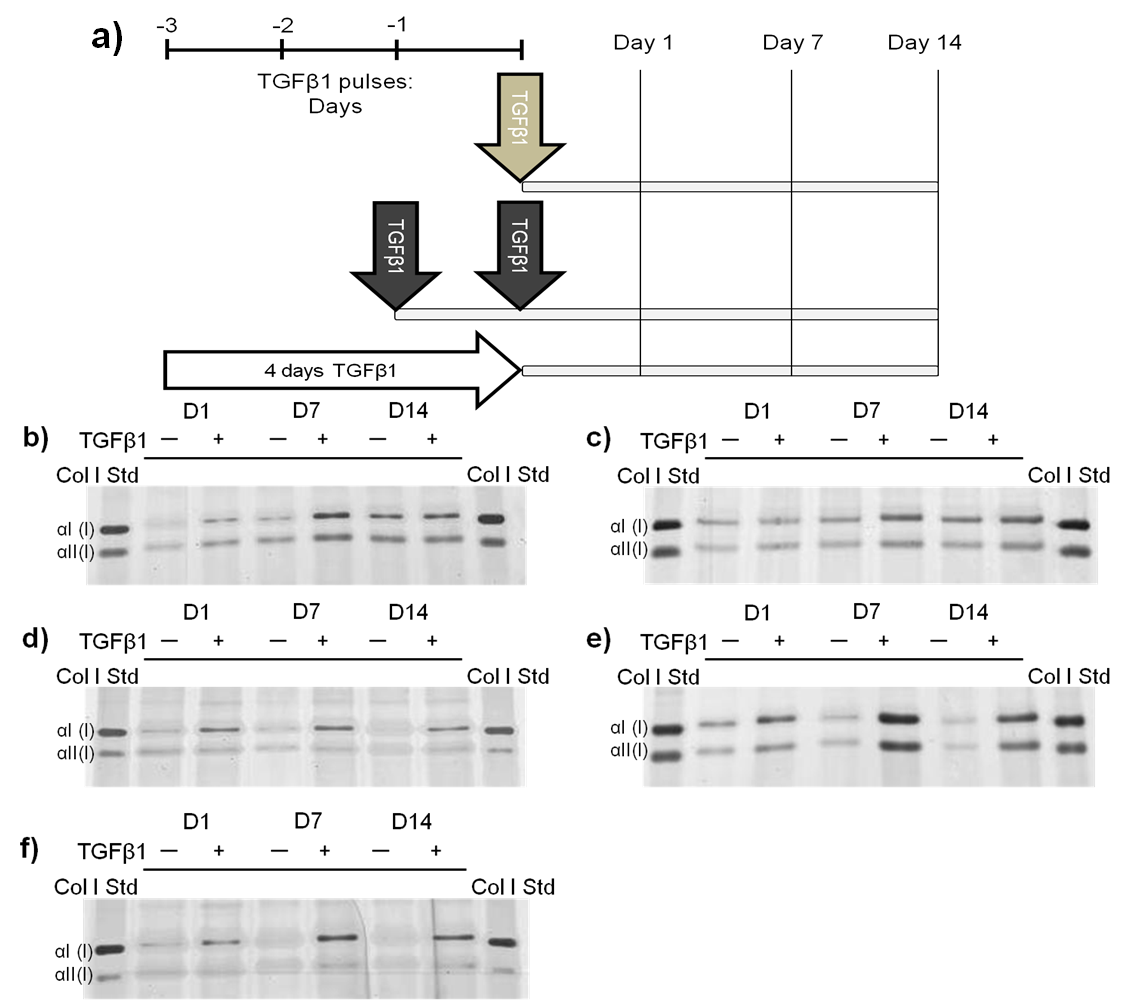

Supplement: Additional file 1: Figure S1 — SDS-PAGE gels comparing short TGF-β1 pulse(s) and 4 days of continuous TGF-β1 treatment. (a) Growth-arrested fibroblasts were treated with or without TGF-β1 according to the cell culture setup comparing single and double TGF-β1 pulses with the traditional 4 days of TGF-β1 treatment. Corresponding silver-stained SDS-PAGE gels for the (b) 0.5 h, (c) 4 h; (d) 2 × 0.5 h, (e) 2 × 4 h; and (f) 4 days of TGF-β1 treatments from which densitometric analysis of the 24 h collagen secretion rate was derived. TGF-β1 transforming growth factor-β1. [file 1755-1536-6-12-S1.tiff]

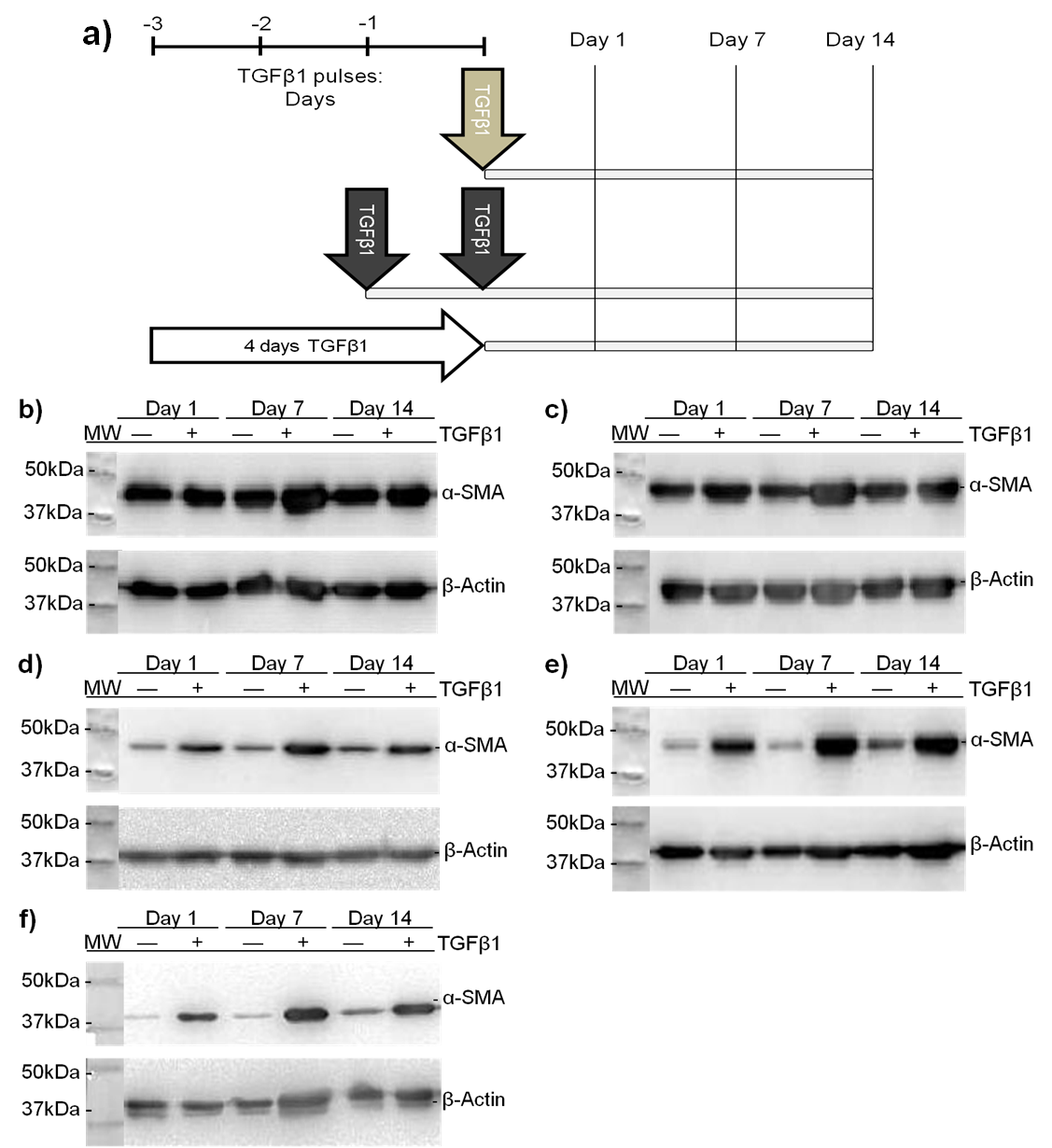

Supplement: Additional file 2: Figure S2 — Immunoblots comparing short TGF-β1 pulse(s) and 4 days of continuous TGF-β1 treatment. (a) Growth-arrested fibroblasts were treated with or without TGF-β1 according to the cell culture setup comparing single and double TGF-β1 pulses with the traditional 4 days of TGF-β1 treatment. Corresponding α-SMA immunoblots for the (b) 0.5 h, (c) 4 h; (d) 2 × 0.5 h, (e) 2 × 4 h; and (f) 4 days of TGF-β1 treatments from which densitometric analysis of α-SMA normalised to β-actin bands was derived. α-SMA, α-smooth muscle actin; TGF-β1 transforming growth factor-β1. [file 1755-1536-6-12-S2.tiff]

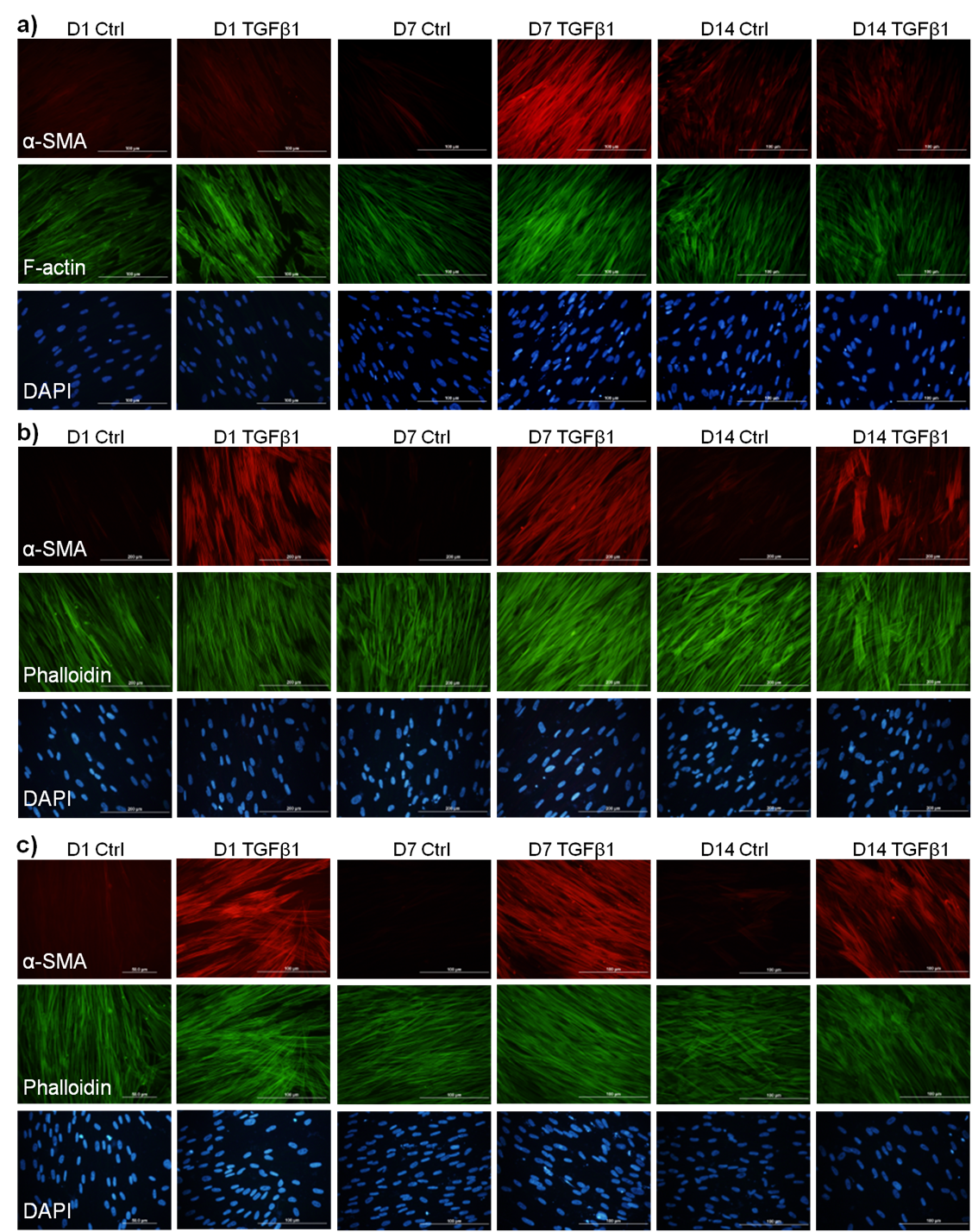

Supplement: Additional file 3: Figure S3 — Immunofluorescence images comparing short TGF-β1 pulse(s) and 4 days of continuous TGF-β1 treatment. Immunofluorescence images showing presence and distribution α-SMA (red); F-Actin (phalloidin, green) and nuclei stained with DAPI (blue) from (a) 4 h; (b) 2 × 4 h TGF-β1 pulsed; and (c) 4 days of TGF-β1-treated cell layers. Scale bars = 200 μM. *P < 0.05 versus respective untreated controls. α-SMA, α-smooth muscle actin; TGF-β1 transforming growth factor-β1. [file 1755-1536-6-12-S3.tiff]

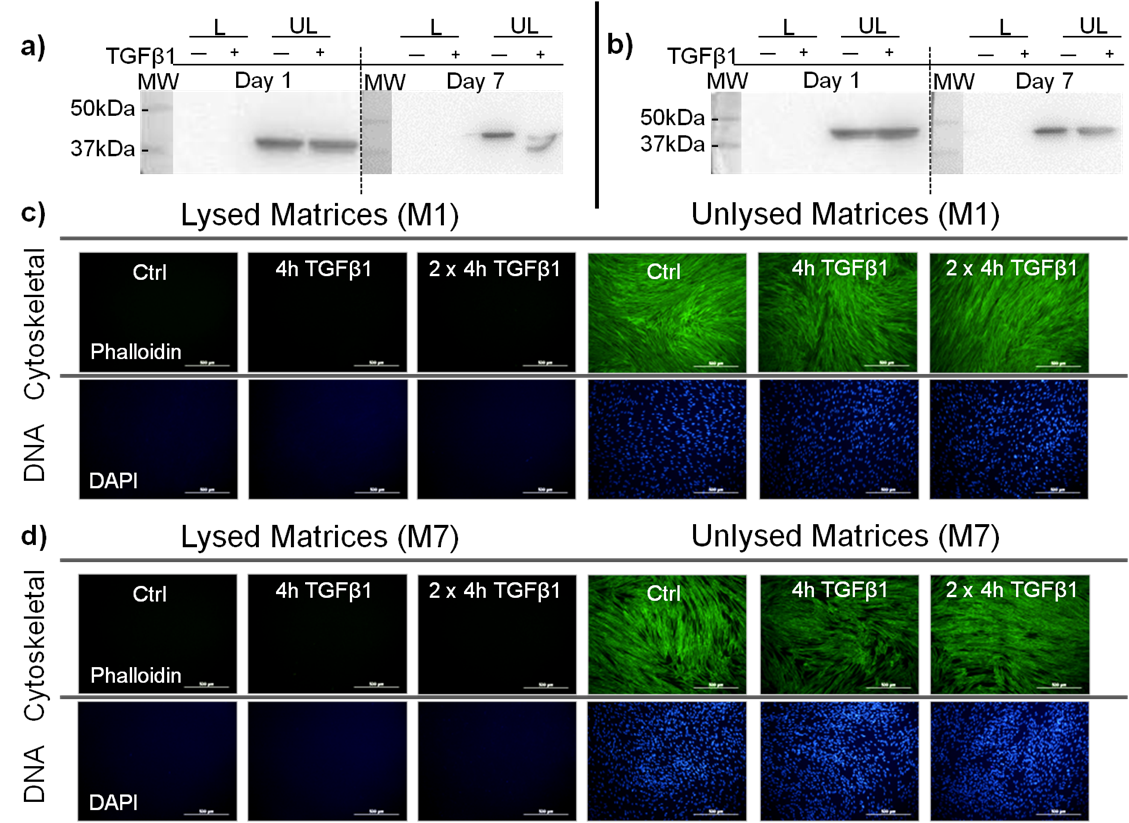

Supplement: Additional file 4: Figure S4 — TGF-β1-pulsed decellularised ECM was free from cellular and matrix residues. The absence of actin and DNA residues was observed in lysed ECM. Representative immunoblots of (a) 4 h; and (b) 2 × 4 h TGF-β1-pulsed ECM. Representative ICC pictures of (e) early M1; and (f) late M7 ECM showing the presence and distribution of cytoskeletal element F-actin (phalloidin, green); and nuclei stained with DAPI (blue). ‘L’ denotes decellularised ECM and ‘UL’ the unlysed ECM (positive matrix control). ECM, extracellular matrix; TGF-β1 transforming growth factor-β1. [file 1755-1536-6-12-S4.tiff]

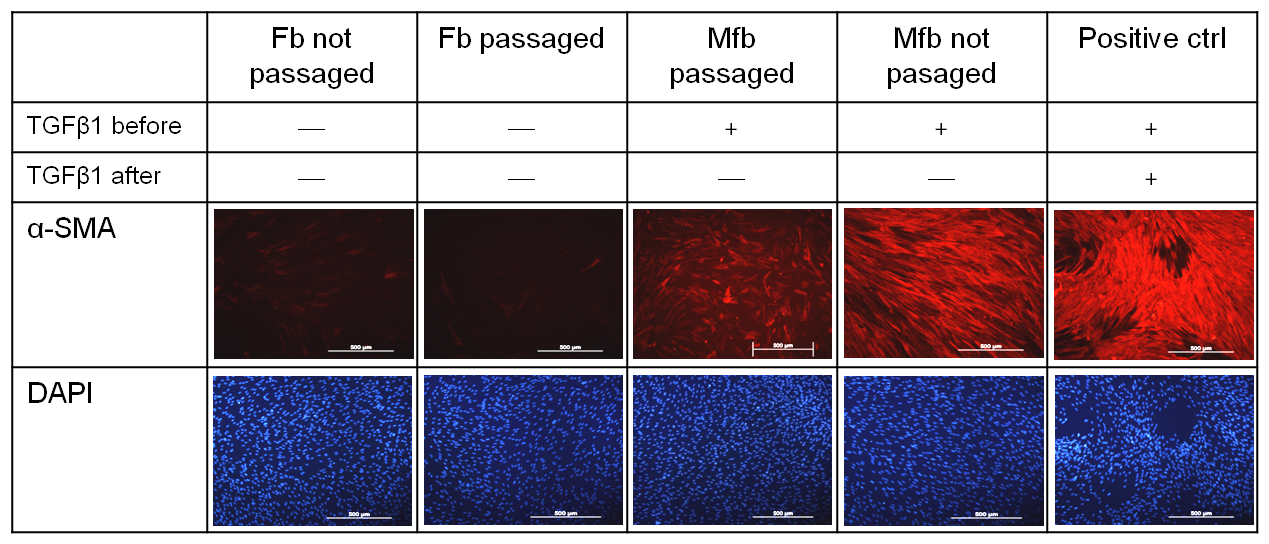

Supplement: Additional file 5: Figure S5 — Trypsin-EDTA attenuated the myofibroblast phenotype. (a) Cell culture setup of TGF-β1-treated myofibroblasts and subsequent sub-culture. Fibroblasts were treated with and without TGF-β1 for 4 days before passaging using trypsin. Myofibroblasts were replated onto TCP. (b) Normalised densitometric SDS-PAGE analysis of the 24 h collagen secretion rate by induced fibroblasts; (c) corresponding silver-stained gel; (d) densitometric analysis of α-SMA immunoblots normalised to β-actin bands; (e) corresponding immunoblot; and (f) immunofluorescence images showing presence and distribution α-SMA (red); F-Actin (phalloidin, green) and nuclei stained with DAPI (blue) from passaged myofibroblasts. Scale bars = 200 μM. *P <0.05 versus respective untreated controls. Data are represented as mean ± SD, calculated from three independent studies in triplicate, and expressed as fold changes over respective controls. α-SMA, α-smooth muscle actin; SD, standard deviation; TCP, tissue culture plastic; TGF-β1 transforming growth factor-β1. [file 1755-1536-6-12-S5.tiff]

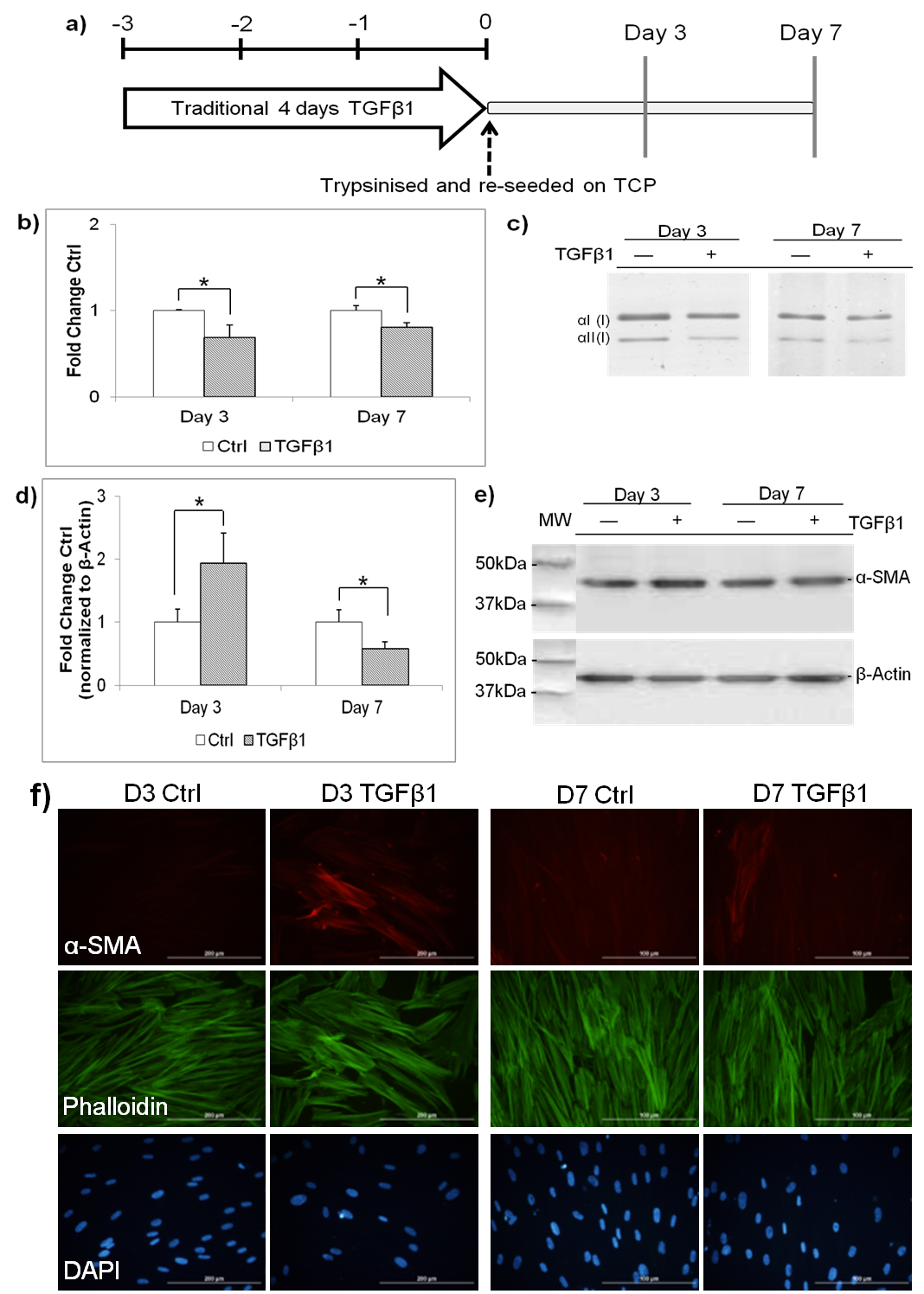

Supplement: Additional file 6: Figure S6 — Dispase passaging of myofibroblasts preserved phenotype but reduced α-SMA expression. Fibroblasts were treated with or without TGF-β1 for 4 days. Thereafter, dispase was employed to passage myofibroblasts and cultures maintained for further 7 days post-replating on TCP. Immunofluoresence images showing the presence and distribution of α-SMA (red); and nuclei stained with DAPI (blue). Scale bars = 500 μM. α-SMA, α-smooth muscle actin; TCP, tissue culture plastic; TGF-β1 transforming growth factor-β1. [file 1755-1536-6-12-S6.tiff]

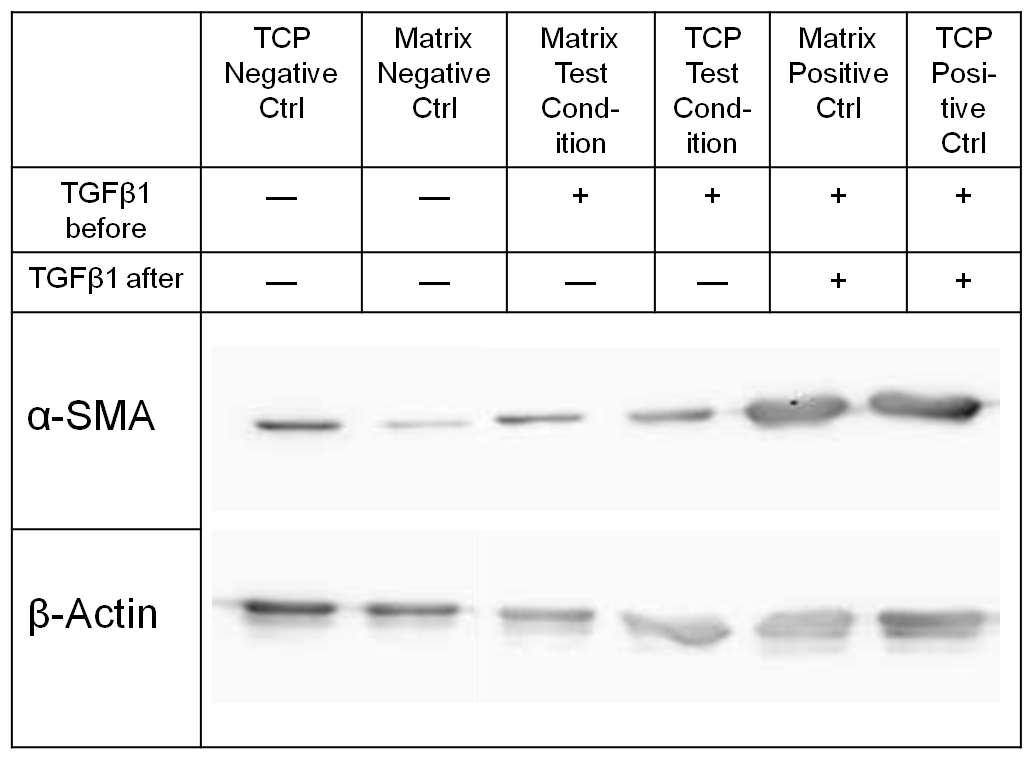

Supplement: Additional file 7: Figure S7 — Immunoblots from cell layers of myofibroblasts reseeded on fibroblast ECM. Corresponding consolidated immunoblot showing the persistence of α-SMA expression after 4-day TGF-β1-treated myofibroblasts were dispase passaged and reseeded onto fibroblast ECM. ECM, extracellular matrix; TGF-β1 transforming growth factor-β1. [file 1755-1536-6-12-S7.tiff]
